# Improving Reactivity of Pumice, Perlite and Farin by Mechanochemical Activation

**DOI:** 10.3390/ma19091702

**Published:** 2026-04-23

**Authors:** Safa Nayır

**Affiliations:** Department of Civil Engineering, Karadeniz Technical University, Trabzon 61080, Türkiye; safanayir@ktu.edu.tr

**Keywords:** pumice, perlite, mechanochemical activation, pozzolanic reactivity, bound water

## Abstract

This study investigated the improvement of the pozzolanic activity of pumice, perlite, and farin through mechanochemical activation (MCA). The properties of the materials were determined by performing XRF, XRD, and particle size and specific surface area analyses. The MCA of three different materials sourced from Türkiye was performed using a planetary ball mill, and their pozzolanic reactivity was systematically investigated. R^3^ test (bound water measurement) and strength activity index (SAI) test were used to evaluate pozzolanic activity. Based on the results, following MCA, the crystal structure was significantly disrupted, particularly in perlite and pumice, and the amount of amorphous phase increased more compared to farin, as confirmed by the decrease in XRD peak intensities. The amount of bound water tended to increase by increasing grinding time and grinding speed. The highest amount of bound water (7.5%) was obtained by grinding the pumice sample at 500 rpm, with ball-to-powder ratio (BPR) of 10 for 60 min. For the same material, the highest activity index (106%) was determined at 500 rpm, with a BPR of 15 and a grinding time of 60 min. In the perlite sample, the highest amount of bound water (7.07%) and the highest strength activity index (98%) were measured in the sample ground at 500 rpm for 60 min with a BPR of 15. In the farin sample, the highest amount of bound water (3.40%) was obtained at 500 rpm for 40 min with a BPR of 15, while the highest strength activity index (71.05%) was observed at 500 rpm for 40 min with a BPR of 10. The results show that the applied MCA process increases the activity of the materials.

## 1. Introduction

In concrete production, cementitious binders and pozzolanic rock materials are used as substitutes for cement due to their various advantages and economic properties. Cementitious binders increase strength and durability in the long term, reducing the amount of clinker in the cement [1,2]. Statistical studies show that the cement industry is responsible for approximately 5–8% of global CO_2_ emissions [3,4,5]. Therefore, reducing the use of cementitious binders or preferring alternative binders makes a significant contribution to reducing CO_2_ emissions, which are one of the main causes of global warming [1,6]. However, the availability of industrial by-products such as fly ash, silica fume, and blast furnace slag is expected to decrease in the future. This situation is increasing interest in natural materials that are available in certain quantities, such as perlite and pumice. However, the use of these natural materials remains limited due to their low pozzolanic reactivity. In this scope, mechanochemical and thermal activation methods are applied to increase the reactivity of both natural and existing binder materials and to improve their performance characteristics [7,8,9,10,11,12].

MCA is defined as a process that can increase the reactivity of a processed material under specific conditions by creating structural irregularities through intense grinding. The primary goal of the MCA is to transfer as much energy as possible from the milling balls to the ground material [13,14]. MCA is considered an environmentally friendly alternative by enabling the activation of materials without the need for high calcination temperatures as in thermal activation. MCA focuses on the processing of natural clay and stone-based materials, aiming to transform these minerals into cementitious or pozzolanic materials. Application examples of the use of mechanochemically activated materials as cementitious binders are also included [15]. A study indicates that mechanical grinding significantly increases the specific surface area of pozzolanic materials. This increase in specific surface area leads to increased reactivity, resulting in significant improvements in both compressive strength and durability. Generally, mechanical grinding is described as an effective, clean and economical method for performance enhancement [16,17].

During the MCA process, the reaction of particles occurs in three stages. A theoretical relationship has been developed between grinding time and the change in specific surface area. The first stage is the Rittinger zone, where the increase in specific surface area is proportional to the grinding time and the interaction mechanism of the particles is not considered. In the Aggregation zone, known as the second stage, the newly formed surface areas do not show a linear relationship with the amount of energy applied. The aggregation of particles is controlled by Van der Waals forces. In the third stage, the Agglomeration zone, the specific surface area decreases, and the particles chemically bond together. Moisture can affect the material at this stage, and this can be reduced with various types of dispersant additives [15,18,19]. In another study, additional information was provided stating that the adhesion of the ground particles to the surface of the jar and grinding balls is described as “caking.” This phenomenon is simply an adhesion situation. This situation interferes with and affects the grinding process. It means that with the accumulation of the first layer, it will continue with the accumulation of successive layers of material that no longer participate in the grinding process. This effect is related to the third stage and should be taken into consideration [20].

During the MCA, very high mixing and energy levels are achieved. This leads to significant changes in the crystal structure of the material. Allotropic phase transitions can occur as a result of increased structural disorder and transformations of crystalline phases, and a significant increase in the chemical reactivity of the material is observed. The fundamental mechanisms by which MCA improves the pozzolanic or hydraulic behavior of the material are associated with these structural and chemical transformations [21,22,23,24]. Furthermore, mechanical stress generated during impacts can break not only chemical bonds in molecules but also strong bonds such as covalent and ionic bonds [25,26]. It is stated that MCA can be considered an environmentally friendly technique by contributing to the reduction of CO_2_ emissions instead of thermally intensive energy-demanding methods. It is also mentioned that it will enable the development of environmentally conscious and sustainable new-generation materials for the construction sector using natural materials and industrial waste [15].

Pumice can exhibit varying degrees of reactivity depending on its cooling rate and aging process. It has been reported that one-part hydraulic cement with modified mineralogical components can be produced by mechanochemically processing a mixture of pumice and other raw materials [27]. Another study showed that an increase in the specific surface area of mechanochemically treated kaolin significantly improved the early age hydration rate and strength development [28]. Another study conducted on pumice with low pozzolanic reactivity investigated the effects of grinding type and duration on particle size and microstructure. According to the results, approximately 6 h of grinding resulted in a significant decrease in particle size. It was also stated that with increasing grinding time, the specific surface area increased, leading to a significant increase in lime consumption at an earlier age [29]. Another study on enhancing the pozzolanic reactivity of clay, marl, and obsidian through mechanochemical means showed that a 20 min grinding process resulted in a decrease in particle size and an increase in surface area. It has been reported that cement mortar mixtures prepared with 20% mechanochemically treated clay and marl reached strength activity index values of 88% and 81% after 28 days. These values are stated to be 37.7% and 24% higher, respectively, compared to standard milled counterparts [30]. In another study investigating the MCA properties of three different natural acidic powders for use in cement, the powders were ground for different durations and in varying quantities. It is stated that substituting pumice, perlite, and granite for cement in traditional mortars may provide advantages in terms of compressive strength. The effect of MCA on compressive strength improvement is reported to be higher at 10% and 20% substitution rates compared to 30% substitution rate in the cement mixture [31].

The current literature contains a limited number of studies on enhancing the reactivity of natural materials and farin; through MCA, most of these studies rely on long grinding times and limited process parameters. Furthermore, the effectiveness of short-term activation conditions, the comparative performance of different natural materials, and determination of optimum process parameters has not been sufficiently addressed. Accordingly, this study aims to systematically reveal the effectiveness of MCA on the pozzolanic activity of three different materials. Since grindability behavior is directly related to the hardness, crystal structure, and mineralogical composition of the materials, optimizing the activation process for each material is critically important. In this scope, a comparative evaluation was carried out considering the effects of key process parameters such as grinding time, speed and ball-to-powder ratio (BPR). Different experimental methods were used to determine pozzolanic activity and reveal the performance relationship between the materials.

## 2. Experimental Study

In the experimental study, a Retsch-PM 100 model planetary mill was used to perform MCA. The milling conditions are given in Table 1. Grinding time, grinding speed, mill type, nature and size of grinding beads, BPR, and jar size are the parameters that affect the MCA.

The variables covered in this study are grinding time, speed, and BPR. The MCA process was evaluated under various conditions by processing samples with different BPR and grinding time (20, 40 and 60 min).

**Figure 1:**
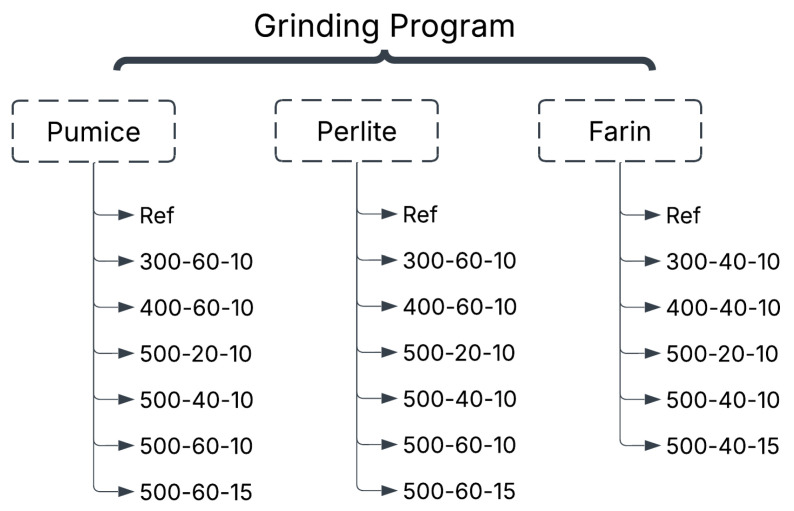
Grinding program in experimental study.

**Figure 2:**
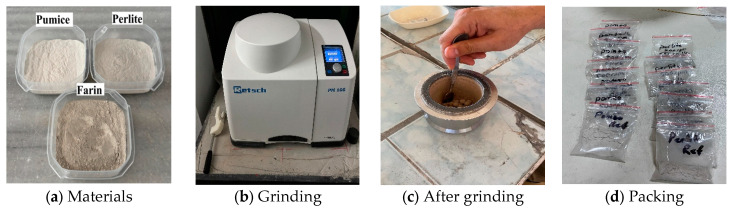
Flowchart of the mechanochemical activation procedure.

Pumice, perlite, and farin were used in the experimental study. Farin is a raw, powdered mixture obtained by mixing and finely grinding limestone and clay in specific proportions. Throughout the article, this raw mixture is referred to as “farin”. Figure 1 schematically presents the mechanical grinding procedure applied to pumice, perlite, and farin samples. In sample naming, the first digit indicates the rotational speed (rpm), the second digit indicates the grinding time (min), and the third digit indicates the BPR. For example, the sample coded 5006010 indicates material ground at 500 rpm, for 60 min, and at a BPR of 10. Similarly, the code 50040-15 indicates grinding conditions performed at 500 rpm, for 40 min, and at a BPR of 15. The “Ref” code represents reference samples that have not undergone any mechanical grinding process. Figure 2 shows a flowchart of the mechanochemical activation procedure in the experimental study.

X-ray diffraction (XRD) patterns of the materials were collected in the range of 2θ° = 5–60°. Specific surface areas of pumice, perlite and farin were carried out using the BET method (Autosorb-iQ2-MP/Kr BET Surface Analyzer, Quantachorme Instruments, Boynton Beach, FL, USA). BET analysis is a method based on the physical adsorption of gases such as nitrogen at low temperatures (77 K) used to determine the specific surface area and pore distribution of solid or powder materials. In this method, adsorption data at different pressures are evaluated using Brunauer–Emmett–Teller (BET) theory to calculate the surface area and obtain information about the pore structure. The particle size distribution of materials was characterized using the Mastersizer 2000 (Malvern Instruments, Malvern, UK) laser particle size analyzer.

Rapid, relevant and reliable (R^3^) test was studied on mortars according to ASTM C1897-20 [32] standard. There are two ways to assess reactivity in this test. The first is to measure heat release using isothermal calorimetry. The second is a simple method that determines the bound water content, which is then calculated and interpreted. Because such isothermal calorimetry devices are not available in all laboratories, the latter method was chosen for this study. A linear relationship has been established between the bound water values obtained in the R^3^ test results of calcined kaolinitic clay and the strength results [33]. In the experimental study, the bound water content of hydrated pastes consisting of binder material, calcium hydroxide, calcium carbonate, potassium sulfate, and potassium hydroxide was determined after curing at 40 °C for 7 days. As specified in the standard, calcium hydroxide, calcium carbonate, potassium sulfate, and potassium hydroxide were combined in ratios that would form a paste in which the soluble ions from these components simulate the pore solution in the Portland cement system. In the experimental study, the ratio of the material to calcium hydroxide is 1:3 by mass. The ratio of the material to calcium carbonate is 2:1 by mass. In addition, a potassium solution was prepared by dissolving 4 g of potassium hydroxide and 20 g of potassium sulfate in 1 L of reactive water (conditioned at 23 ± 3 °C). The ratio of potassium solution to solids (sum of materials, the calcium hydroxide, and the calcium carbonate) is 1.2 by mass.

**Figure 3:**
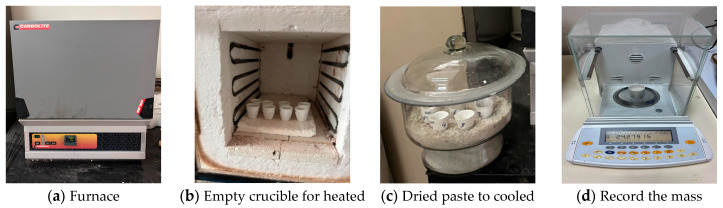
Experimental procedure followed to determine the amount of bound water.

The calculation method was performed following this sequence: First, the oven was heated to 350 ± 10 °C. An empty porcelain crucible was placed in the pre-heated oven at 350 ± 10 °C for 1 h ± 10 min. Then, the empty porcelain crucible was cooled to room temperature in an empty dryer. The porcelain crucible containing the dried paste was placed in the pre-heated oven at 350 °C for 2 h 10 min. The heated paste was cooled appropriately. The percentage of bound water was calculated. Figure 3 shows the experimental procedure followed to determine the amount of bound water.

**Figure 4:**
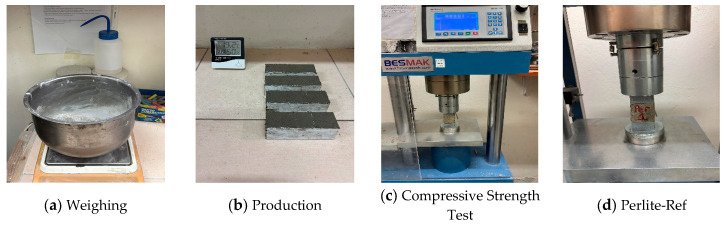
Flowchart of experimental procedure for determining strength activity index.

SAI is an indirect method used to evaluate pozzolanic activity based on the comparative compressive strength of mortars. The evaluation is carried out based on a comparative relationship established with the reference sample. SAI is the percentage expression of the compressive strength of the additive mortar to the compressive strength of the reference mortar containing 100% cement. In the experimental study, pozzolanic reactivity was evaluated using the strength activity index method based on compressive strength test of mortars according to the ASTM C618 standard [34]. This method is an indirect assessment approach. Cube-shaped mortar samples, each measuring 4 × 4 × 4 cm^3^, were prepared for use in compressive strength tests. Three samples were prepared. Sample production and testing were carried out in accordance with the principles specified in standard. A water/cement ratio of 0.50 was selected to ensure a homogeneous mixture in the mortar mixtures. CEM I 42.5 R type cement used. Standard sand was used as the aggregate component of the mixture, thus ensuring that the experimental results reflect only the effect of the additive, independent of the different mixture parameters. Mortar samples were prepared to contain one part cement, three parts standard sand, and half a part water by weight. Mortar samples were casted under laboratory conditions (18–20 °C temperature and 50–55% relative humidity). After casting, mortars were kept in a standard curing for 28 days (20 ± 2 °C water). All prepared mixtures were cast into molds, with three specimens produced from each mixture and compacted using a shaking table in the experimental study. After being removed from the molds, the samples were held for a specified period under standard conditions. Compressive strength tests were conducted after the curing period was completed. A loading rate of 0.6 MPa/s was selected during the experiments. Figure 4 shows the flowchart of the experimental procedure for determining strength activity index.

XRF analyses of the pumice, perlite and clay powder used were carried out in the central laboratory and are given in Table 2.

## 3. Result and Discussion

Pumice, perlite, and farin were subjected to mechanical activation at different speeds, durations and BPR. X-ray diffraction (XRD) analysis, particle size and BET analyses were performed on the mechanically activated sample. Particle size and BET analyses tests evaluated the preparation of mortar samples for use in strength activity tests. Additionally, analyses of bound water were conducted in accordance with the relevant standards to assess the chemical reactivity of the materials. These data obtained were used comparatively as key parameters in determining the reactivity of the mechanically activated samples. The results of the study are discussed in detail in the following section.

### 3.1. XRD Analyses

**Figure 5:**
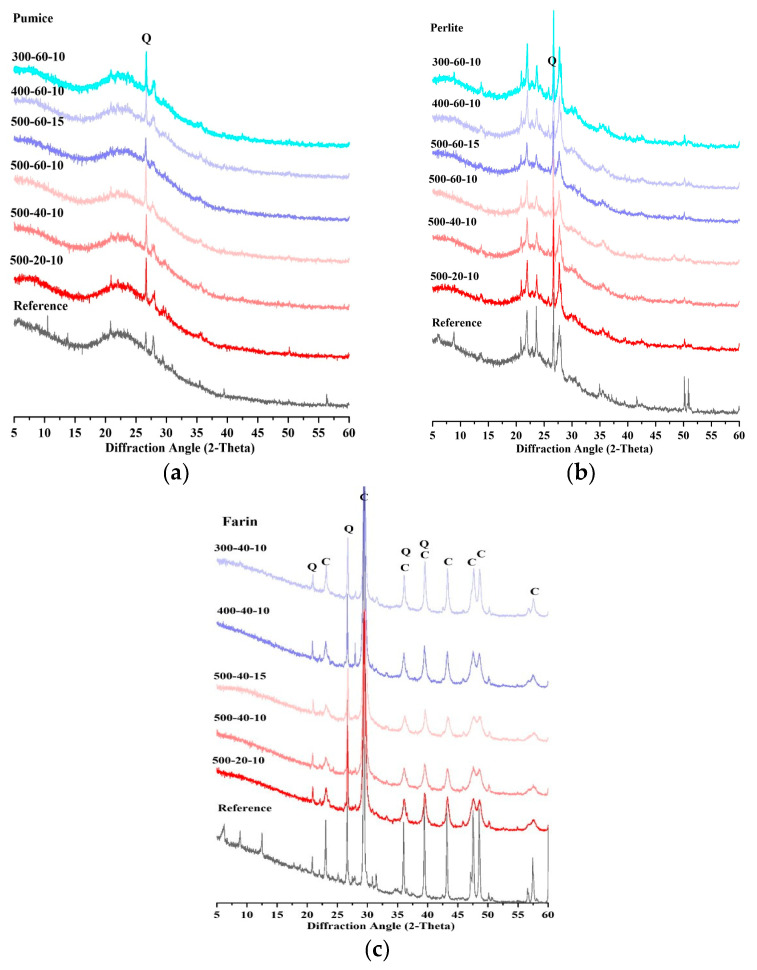
XRD patterns of pumice (**a**), perlite (**b**) and farin (**c**) after MCA.

XRD analyses were performed on samples to examine the structural changes occurring during mechanical activation of the three different materials studied. Figure 5 shows the XRD patterns of the three different samples depending on grinding time, speed and BPR.

According to Figure 5, a continuous broadening of the diffraction peaks is observed as the grinding time increases due to particle fragmentation and plastic deformation. The formation of amorphous phases after MCA increases the disorder in the crystal structure, which is observed as a decrease in the intensity of the XRD peaks. This type of behavior is thought to be a characteristic result of high-energy ball milling processes. Similar results are highlighted in other studies on mechanical activation [35,36,37]. Comparison and evaluation of XRD patterns show that the degree of crystallinity in the samples determines their structural fineness and resistance to grinding. More crystallinities indicate the least elongated defects possible in the material structure, such as stacking faults and dislocations; in other words, greater crystallinity leads to greater strength. The reduction in crystallite size during MCA increases the reactivity of the material. The amorphous phase is normally considered the most reactive state for a material [35]. Perlite and pumice show a rapid decrease in the intensity of reflections in MCA patterns. This indicates that structural changes occur during the MCA.

After grinding the pumice, a significant decrease in intensity and a broadening of the peaks is observed, especially in the 22–30° range. Peak broadening can be associated with irregularities in the crystal structure, primarily the reduction in crystal size. This indicates that the crystal order is partially disrupted and, consequently, the amount of amorphous phase increases. At a constant grinding speed of 500 rpm, it was determined that the peak intensities gradually decreased as the grinding time increased. In addition, the broad-based amorphous hump observed in the 20–35° range became more pronounced. This result reveals that the structure becomes more amorphous with MCA progression. The intensity of the main and secondary peaks belonging to quartz also tends to decrease as the grinding time and BPR increase. Silica, a reactive phase, is formed as a result of the decomposition of quartz. It is thought that a significant amount of reactive silica is formed as a result of the grinding process. Another study confirms that the increase in the degree of amorphization, which is the amount of reactive phase in a material, is affected by the grinding time. Consequently, the grinding process leads to the breakdown of the pumice’s crystalline structure and the formation of an amorphous silica phase [29].

When the XRD patterns of the perlite sample were examined after MCA, it was determined that the quartz peak, observed especially around 26.6°, showed a tendency to decrease in intensity compared to the reference sample. Furthermore, a broad amorphous hump originating from the glassy (amorphous) structure of perlite is noticeable in the 20–30° range. This indicates that the perlite contains a significant amount of amorphous phase and that the crystalline order is partially disrupted during the MCA process. In the farin sample, the decrease in peak intensities after MCA was more pronounced, especially in the peaks belonging to the quartz and calcite phases. When the same grinding time and BPR were kept constant, the decrease in peak intensities was observed to be more pronounced with increasing rpm speeds. This suggests that high grinding energy has a stronger disruptive effect on the crystalline structure, leading to partial amorphization of the crystalline phases.

A decrease in peak intensity is observed, particularly noticeable in the sample exposed to 500 rpm. This can be attributed to the disruption of the crystal structure and a decrease in the crystal phase ratio. When comparing the decrease in densities, pumice and perlite appear to be more amorphous materials compared to farin. Another study indicates a significant decrease in densities and structural disruptions in the results of XRD analyses of the samples after MCA [31].

### 3.2. Particle Size and Specific Surface Area of Materials

**Figure 6:**
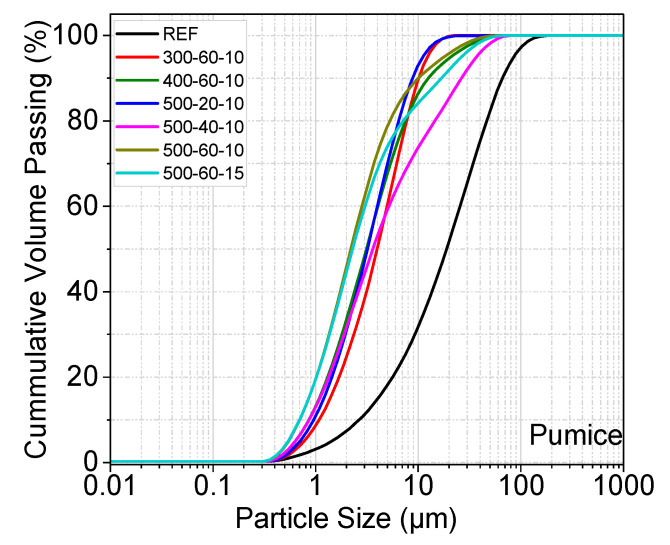
Particle size distribution of reference and post-mechanical activation materials according to grinding time and ball/powder ratio.

**Figure 7:**
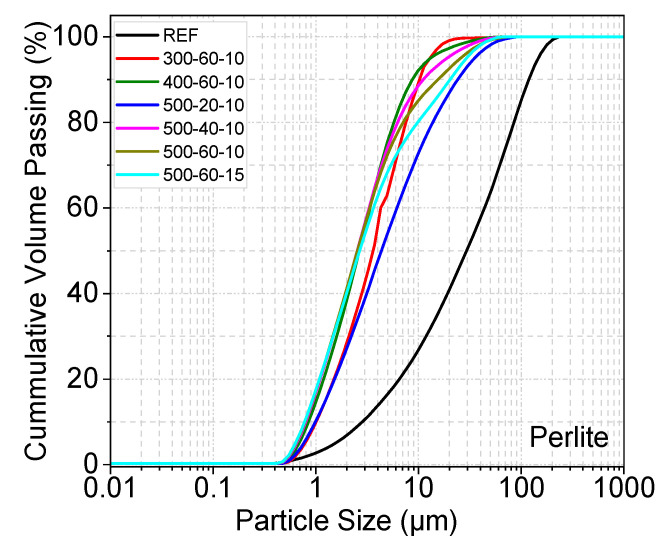
Particle size distribution of reference and post-mechanical activation materials according to grinding time and ball/powder ratio.

**Figure 8:**
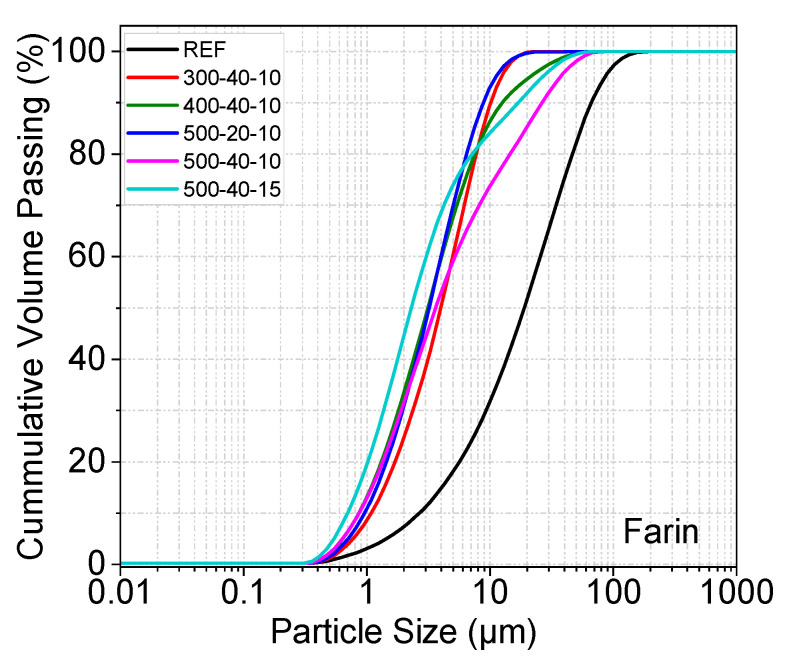
Particle size distribution of reference and post-mechanical activation materials according to grinding time and ball/powder ratio.

Figure 6, Figure 7 and Figure 8 show the particle size distribution of the three different samples after MCA. The reference sample was not subjected to the grinding process. In all samples except the last one, the ball/powder ratio is 10. After MCA, the materials showed a certain degree of particle size reduction compared to the reference sample. A shift to the left is noticeable in the curve after grinding. In pumice and perlite samples, the finest particles were observed in samples numbered 500–60–10 and 500–60–15. The shift to the left was less pronounced at lower speeds and for shorter durations. The fine and more homogeneous structure formed after MCA can be associated with the reactivity of the material. In the farin samples, the finest particle was observed in samples coded 500–40–10 and 500–40–15. Furthermore, a decreasing trend in fracture rate was observed as the milling process progressed.

After MCA, the D_50_ values (median diameter) of the pumice samples were determined as 4.063 µm, 3.18 µm, and 2.47 µm for samples coded 300–60–10, 400–60–10, and 500–60–10, respectively. The D_50_ value of the sample coded 500–60–15 was slightly lower than that of the 500–60–10 sample, at approximately 2.34 µm. D_50_ values of the perlite samples were determined as 3.69 µm, 2.71 µm, and 2.58 µm for samples coded 300–60–10, 400–60–10, and 500–60–10, respectively. The D_50_ value of the perlite sample coded 500–60–15 was 2.40 µm. A decrease in pumice and perlite particle size occurred with increasing grinding speed and BPR.

A decrease in farin particle size occurred with increasing grinding speed and BPR. After MCA, the D_50_ value of the farin samples was determined as 7.75 µm, for samples coded 300–40–10. D_50_ value of the reference farin samples was determined as 19.56 µm.

**Figure 9:**
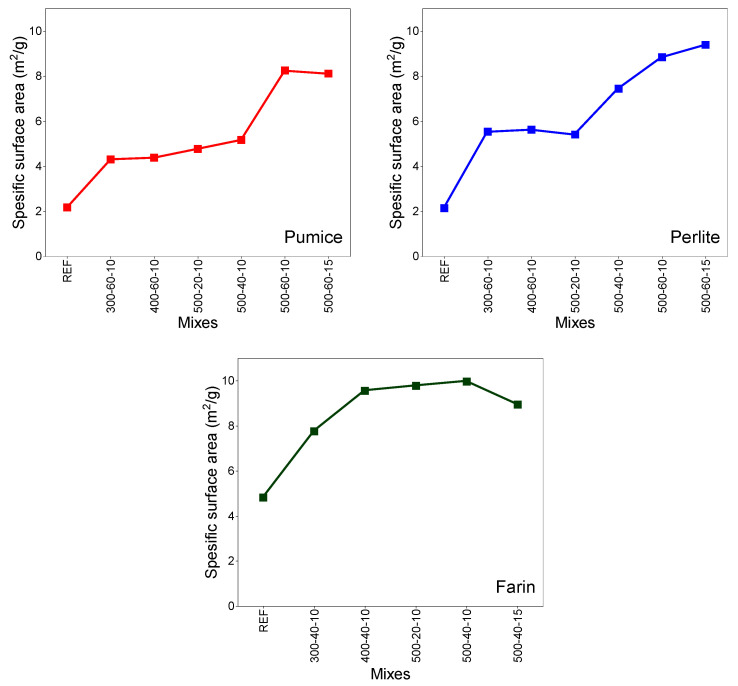
Specific surface area of materials after MCA.

Figure 9 shows the changes in the BET specific surface areas of the three different samples with grinding time. Analysis results show a significant increase in the BET surface areas of the materials as grinding time increases. This is attributed to the reduction in particle size and the resulting expansion of specific surface areas during the grinding of perlite, pumice, and farin. Additionally, in different studies, it is stated that the mechanochemical reaction increases finesses, reduces particle size, causes structural disorder and amorphization, and increases chemical reactivity [13,38,39]. A significant increase in specific surface area was observed with increasing grinding speed and duration.

While the reference specific surface area for pumice was 2.17 m^2^/g, these values reached 4.31, 4.38, and 8.25 m^2^/g for samples coded 300–60–10, 400–60–10, and 500–60–10, respectively. It was particularly observed that the specific surface area increased with increasing duration at a constant speed of 500 rpm. Compared to grinding times of 20 and 40 min, the increase observed at 60 min of grinding was more pronounced. This situation can be attributed to the fact that, under the influence of mechanical energy, the grains break and transform into a finer morphology. Conversely, increasing the BPR resulted in a partial decrease in specific surface area. Indeed, while the specific surface area of the pumice sample ground at 500 rpm for 60 min with 10 BPR was 8.25 m^2^/g, increasing the BPR to 15 at the same speed and duration led to a decrease in the specific surface area. This decrease can be attributed to the fact that the increase in the BPR may create excessive impact on the material, leading to agglomeration. The agglomeration effect is also consistent with the particle size distribution results. The D_50_ value of the sample coded 500–60–15 is slightly higher compared to the sample coded 500–60–10. In another study on MCA study with basaltic fines shows that increasing grinding time leads to an increase in specific surface area, but subsequent decreases due to stabilization or particle agglomeration [40].

The specific surface area of the reference perlite sample was 2.14 m^2^/g, while under the 300–60–10, 400–60–10, and 500–60–10 conditions, these values were determined as 5.53 m^2^/g, 5.62 m^2^/g, and 8.84 m^2^/g, respectively. When evaluated in terms of grinding time, the increase in surface area was much more pronounced at 60 min compared to 20 and 40 min. While the specific surface area was measured as 8.84 m^2^/g under the 500–60–10 condition, increasing the BPR from 10 to 15 under the same speed and time conditions increased this value to 9.39 m^2^/g. This indicates that increasing the BPR in perlite increases grinding efficiency and consequently reduces particle size. This result shows that increasing the BPR in perlite provides higher grinding efficiency and leads to a greater reduction in particle size. Therefore, perlite exhibited behavior that responded positively to a high BPR.

The specific surface area of the reference farin sample was 4.82 m^2^/g, while under the 300–40–10, 400–40–10, and 500–40–10 conditions, these values were determined as 7.76 m^2^/g, 9.56 m^2^/g, and 9.97 m^2^/g, respectively. The increase in specific surface area in the farin sample when milling speed changed from 300 rpm to 400 rpm was more pronounced than the increase observed when milling speed changed from 400 rpm to 500 rpm. This suggests that after a certain speed, the additional energy applied does not translate proportionally to the surface area, indicating that the system exhibits partial saturation behavior. Furthermore, it was determined that the percentage increase in surface area with increasing grinding time remained limited. This indicates that new surface formation slows down during prolonged grinding. As in the pumice sample, a decrease in specific surface area was observed when the BPR was increased from 10 to 15 for 40 min at 500 rpm. This decrease can be attributed to agglomeration.

**Figure 10:**
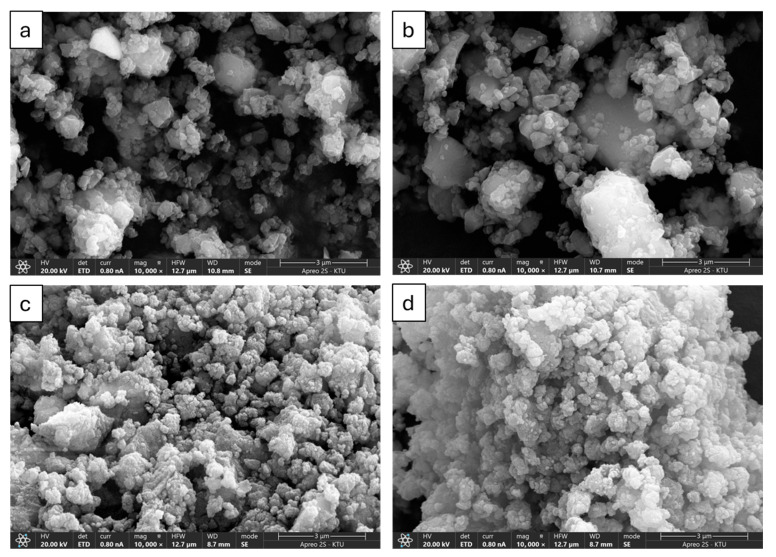
SEM images of pumice (**a**) 500–60–10, (**b**) 500–60–15 and farin (**c**) 500–40–10, (**d**) 500–40–15 samples, at 10,000 magnitude.

Figure 10 shows SEM images of the effect of increasing the BPR from 10 to 15 on particle morphology for both the pumice (Figure 10a,b) and farin (Figure 10c,d) samples. When the BPR is 10 (Figure 10a,c), the particles appear relatively finer and more homogeneously distributed, indicating an effective size reduction and increase in specific surface area due to MCA. However, when the BPR is increased to 15 (Figure 10b,d), a change in morphology is observed. The particles tend to form larger, denser clusters, which are associated with agglomeration. These agglomerates may result from the increased collision frequency and energy input during grinding, which promotes particle sourcing and re-bonding rather than further fragmentation. This trend is consistent for both the pumice and farin samples, demonstrating that higher BPR does not necessarily improve fineness or reactivity due to the dominance of agglomeration mechanisms.

### 3.3. Bound Water Test

The pozzolanic activity index of pumice, perlite, and farin was evaluated using the bound water method, using the ASTM C1897-20 [32]. standard, also known as the R^3^ test. This standard shows that it contributes to the evaluation of the chemical reactivity of natural and artificial pozzolans in a shorter time, with higher accuracy and more efficiently [33,41].

The bound water content for each material used was determined by mixing the material with Ca(OH)_2_, KOH, K_2_SO_4_, CaCO_3_, and deionized water. The respective mixture was prepared and stored in closed containers at 40 °C for seven days. Care was taken to ensure that the paste had dried. After seven days of reaction, the crushed hydrated samples were dried in an oven at 105 °C to achieve constant weight. Finally, the dried samples were heated at 350 °C for 2 h, and the amount of chemically bound water content was determined by considering the weight differences. This process was repeated for all the mixtures.

**Figure 11:**
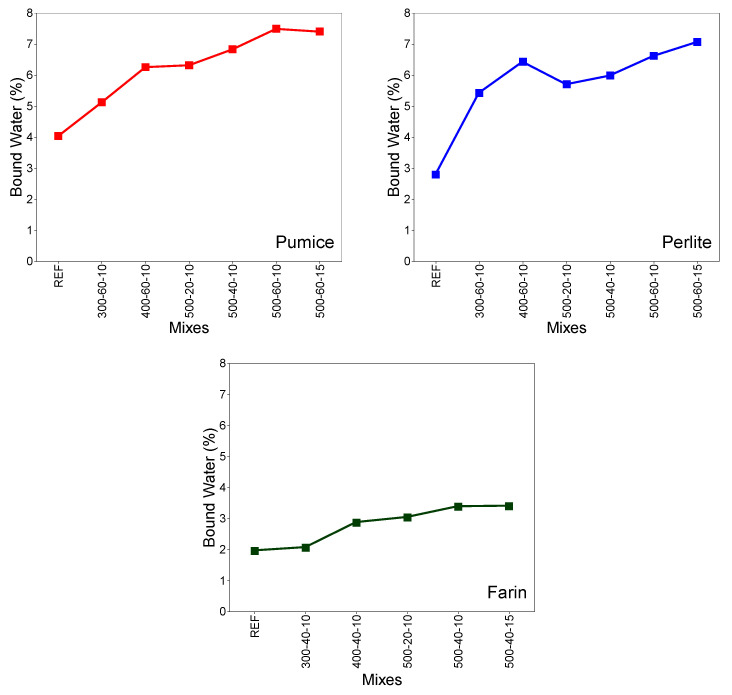
Bound water test results.

Figure 11 shows the bound water percentages calculated within ASTM C1897-20 [32]. A higher amount of bound water generally indicates higher pozzolanic activity. This condition can also be associated with strength development [33]. Experimental studies on pumice samples revealed that increasing the grinding speed resulted in a significant increase in the amount of bound water, regardless of time. Similarly, when the grinding speed was held constant, the amount of bound water tended to increase with increasing processing time. While the bound water content of the reference sample was approximately 4%, this ratio reached approximately 7.5% in the pumice sample milled at 500 rpm for 60 min. Furthermore, the amounts of bound water obtained between 500 rpm and 60 min in systems with a ball/powder ratio of 10 and 15 were found to be quite similar, indicating that the ball/powder ratio has a limited effect on the development of bound water within this range. This increase in the amount of bound water indicates that the pozzolanic activity of the pumice increases due to the refinement and increased reactivity achieved by grinding. A different study investigated the binding material activity of basalt fines. The activity of the sample after MCA was investigated using the R^3^ test. Similarly, it was determined that the reactivity of the material increased by increasing grinding time and ball/powder ratio. X-ray diffraction analysis indicated partial amorphization in the powder sample [40]. A different study evaluating the R^3^ chemical reactivity in various traditional and new-generation pozzolans reported a strong correlation between heat flux measured by isothermal calorimetry and the bound water content of the pozzolans. The study examined commonly used pozzolanic materials such as metakaolin, silica fume, fly ash, and slag [41]. A study examining the validity of the R^3^ reactivity test on different material types reported that when comparing the R^3^ test results with the compressive strength improvement of mortars, all conventional mineral admixtures exhibited a similar correlation trend [42].

Experimental studies conducted on perlite samples revealed that, as in pumice, increasing grinding speed resulted in a significant increase in the amount of bound water, independent of time. Similarly, when grinding speed was held constant, the amount of bound water tended to increase with increasing processing time. While the bound water content of the reference sample was approximately 2.80%, this ratio reached approximately 6.6% in the pumice sample milled at 500 rpm for 60 min. Furthermore, in systems with a ball/powder ratio of 10 and 15, the amounts of bound water obtained at 500 rpm and 60 min were approximately 6.6% and 7%, respectively. It was observed that the effectiveness of the change in the ball/powder ratio was more pronounced in perlite; conducting it on the farin samples revealed that increasing grinding speed resulted in an increase in the amount of bound water, independent of time. Similarly, when grinding speed was held constant, the amount of bound water tended to increase with increasing processing time. While the bound water content of the reference sample was approximately 1.96%, this ratio reached approximately 3.38% in the farin sample milled at 500 rpm for 40 min. Furthermore, in systems with a ball/powder ratio of 10 and 15, the amounts of bound water obtained at 500 rpm and 40 min were approximately 3.38% and 3.40%, respectively.

In general, the amount of bound water in the perlite and pumice samples after MCA ranged from 3% to 8%, while the bound water content in the farin samples remained quite low, varying between approximately 2% and 3.4%. This can be attributed to the fact that farin has a higher crystalline phase content, resulting in a limited increase in reactivity after MCA.

**Figure 12:**
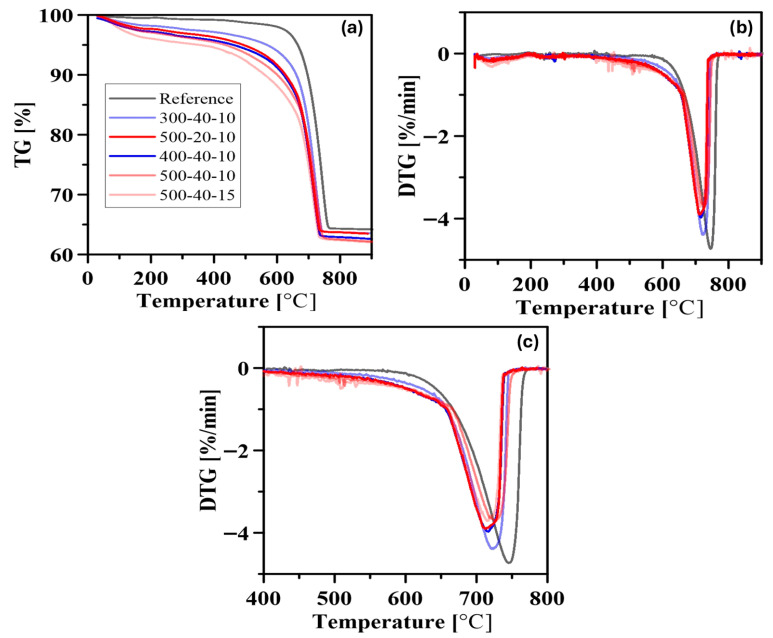
TG (**a**) and DTG (**b**) and 400–800 °C DTG (**c**) curves of the farin samplesobtained from TGA.

The thermogravimetric analysis (TGA) results are shown in Figure 12. The TG-DTG results indicate that the MCA process caused limited structural changes in the farin sample. Figure 12c specifically shows the comparison between 400 and 800 °C. It was observed that the decarbonation peak shifted to lower temperatures compared to the reference sample, along with increasing grinding speed and time. This suggests a decrease in the size of the calcite crystal and partial structural degradation. The decrease in the calcite peak in the XRD analysis in Figure 5c also supports this finding.

### 3.4. Strength Activity Index

Mortar mixtures were designed after MCA of pumice, perlite, and farin under different rpm speeds, duration and BPR. The mortar mixtures were produced by replacing 30% of the cement with these activated materials. In the next stage, the compressive strengths of the samples were measured after a 28-day curing period, considered the most important characteristic. A reference mortar sample consisting only of cement, sand, and water was also produced for comparison. Three compressive strength results were obtained for each series, and the average value was determined; calculations were performed based on this average. The 28-day strength activity index values of the mortar samples were determined according to the ASTM C618 standard [34]. The compressive strength of the reference mortar sample prepared with 100% Portland cement was measured as 43.5 MPa. Samples that did not undergo MCA and in which 30% of the cement was replaced with the relevant material (pumice, perlite, farin) were designated with the code “Ref”. In SAI comparisons, the ultimate reference is a mixture containing 100% cement.

**Figure 13:**
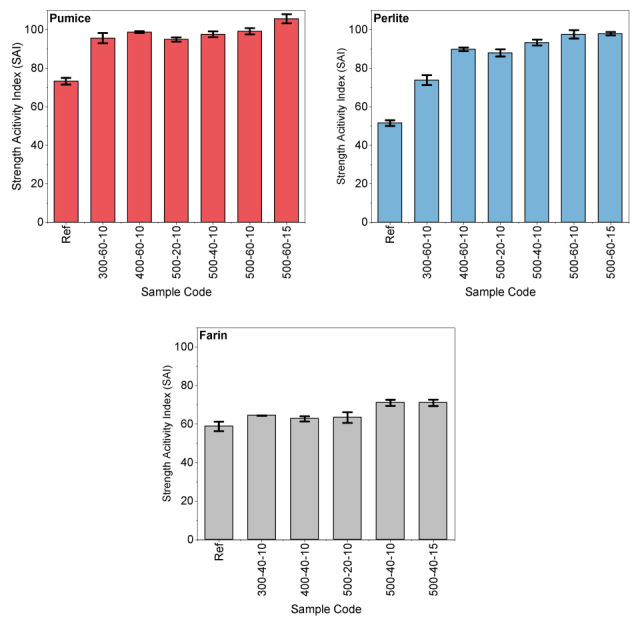
Strength activity index (SAI) results.

Figure 13 shows the SAI results for the mixtures. The compressive strength of the mortar produced with the pumice sample without MCA was determined as 31.87 MPa, while the lowest strength value obtained after MCA was 41.30 MPa. This corresponds to an increase in strength of approximately 30%. In pumice samples, an increase in strength values was observed depending on the increase in grinding speed. The highest strength activity index was determined as 105% in the sample with a BPR of 15 and grinding speed at 500 rpm for 60 min. This result is also consistent with the bound water results obtained from the R^3^ test. In the pumice reference sample, the SAI remained at approximately 73%. The strength activity indices of samples coded 500–20–10, 500–40–10, and 500–60–10, which were subjected to MCA at the same grinding speed but for different durations, were determined to be approximately 95%, 98%, and 99%, respectively.

The replacement of mechanically activated powders generally improved the 28-day compressive strength compared to the reference sample. The compressive strength of the mortar produced with the perlite sample without MCA was determined to be 22.42 MPa, while the lowest strength value obtained after MCA was 32.13 MPa. The highest activity index in the perlite sample was achieved at 98% in the sample with a BPR of 15 at a speed of 500 rpm for 60 min. The strength activity index showed an increasing trend depending on the grinding time at a speed of 500 rpm. The SAI results obtained by increasing the grinding time of the perlite sample from 20 to 60 min, independent of speed and BPR, were consistent with the surface area and bound water results. The strength activity indices of the samples coded 500–20–10, 500–40–10, and 500–60–10, which were subjected to MCA at the same grinding speed but for different durations, were determined to be approximately 88%, 93%, and 98%, respectively. In a different study, it was reported that SAI could reach values up to 120% with the MCA process [43].

In the farin sample, grinding times of 40 min at 300 and 400 rpm and 20 min at 500 rpm resulted in partial strength improvement; however, in other samples, the SAI remained below 70%. These low SAI values observed in the farin samples are also consistent with the R^3^ test results. This low SAI value also coincides with the XRD analyses and can be attributed to the high calcite content and limited presence of amorphous silica in the material. Due to the calcite phase not actively participating in pozzolanic reactions, the strength improvement remained more limited compared to the pumice and perlite samples.

## 4. Conclusions

The effect of MCA (under different grinding speeds, times and BPR) on the improving of pozzolanic reactivity of the materials was investigated using SAI and R^3^ testing methods; the findings were correlated with XRF, XRD, BET and particle size analysis results. Conclusions based on the results obtained from the study are summarized below.

(1) Pumice, perlite, and farin underwent significant plastic deformation and structural changes during intensive MCA at different milling times, speeds and BPR. Pumice and perlite have the potential to be converted into more chemically reactive building materials.
(2) It was revealed that the cementitious properties of the three different materials increased after MCA and that the pumice and perlite materials can be used as a substitute for cement.
(3) The crystal structure was significantly disrupted, particularly in perlite and pumice, with the amount of amorphous phase increasing more compared to farin, which was confirmed by a marked decrease in XRD peak intensities.
(4) MCA increased the surface area in all materials compared to the reference sample; however, this effect varied depending on the mineralogical structure of the material, grinding time and BPR.
(5) The amount of bound water tended to increase by increasing grinding time and grinding speed. The highest amount of bound water was obtained in the pumice sample ground at 500–60–10 with 7.5%, while the lowest amount of bound water was observed in the farin sample ground at 300–40–10 with 2.06%.
(6) MCA significantly improved compressive strength development, resulting in SAI values exceeding 70% after 28 days in both the pumice and perlite samples. The highest activity, at 106%, was obtained in the pumice sample coded 500–60–15. The farin samples coded 500–40–10 and 500–40–15 had a strength activity index above 70%. The results show that the applied MCA process increases the activity of the materials.

Mechanochemical activation offers significant potential for improving material performance and providing new opportunities for the cement and construction industry. However, further research and effort are needed to address the disadvantages of high energy consumption during the activation process and to ensure widespread adoption of this method in the concrete industry.

## Figures and Tables

**Table 1:** Milling condition in the experimental study.

Material input size	<10 mm
Ball-to-powder weight ratio	10 or 15
Media type	Stainless steel
Ball weight (g)	540
Ball diameter (mm)	10
Number of balls	74
Components of material	Tungsten carbide
Media corrosion	Insignificant
Mill speed (rpm)	300, 400 or 500

**Table 2:** Chemical composition of materials.

Composition (%)	Pumice	Perlite	Farin
SiO_2_	71.39	72.44	14.88
Al_2_O_3_	13.27	13.70	5.01
Fe_2_O_3_	2.63	2.00	4.83
CaO	1.78	1.80	70.93
MgO	-	0.47	2.05
Na_2_O	4.01	4.25	-
K_2_O	6.51	5.13	1.12
SO_3_	0.01	-	0.20
MnO	0.09	0.07	-

## Data Availability

The original contributions presented in the study are included in the article, further inquiries can be directed to the corresponding author.

## Abbreviations

The following abbreviations are used in this manuscript:

MCA	Mechanochemical activation
BPR	Ball-to-powder ratio
SAI	Strength Activity Index
XRD	X-ray diffraction
